# Synergism of fermented feed and ginseng polysaccharide on growth performance, intestinal development, and immunity of Xuefeng black-bone chickens

**DOI:** 10.1186/s12917-023-03859-y

**Published:** 2024-01-06

**Authors:** Jie Liu, Huan Wang, Junyi Luo, Ting Chen, Qianyun Xi, Jiajie Sun, Limin Wei, Yongliang Zhang

**Affiliations:** 1 Sanya Institute, Hainan Academy of Agricultural Sciences (Hainan Experimental Animal Research Center), Sanya, 572000 Hainan China; 2 Institute of Animal Husbandry and Veterinary Medicine, Hainan Key Laboratory for Tropical Animal Breeding and Disease Research, Hainan Academy of Agricultural Sciences, Haikou, 571100 Hainan China; 3 Guangdong Provincial Key Laboratory of Animal Nutrition Control, College of Animal Science, South China Agricultural University, Guangzhou, 510642 China

**Keywords:** Ginseng polysaccharide, Growth performance, Immune parameters, Microbial fermented feed, Synergistic effect

## Abstract

Microbial fermented feed (MF) is considered a valuable strategy to bring advantages to livestock and is widely practiced. Oral supplementation of Ginseng polysaccharide (Gps) eliminated weight loss in chickens following vaccination. This study investigated the effects of the combined use of Gps and MF on growth performance and immune indices in Xuefeng black-bone chickens. A total of 400 Xuefeng black-bone chickens at the age of 1 day were randomly assigned to four groups. Normal feed group (Control group), ginseng polysaccharide (200 mg/kg) group (Gps group), microbially fermented feed (completely replace the normal feed) group (MF group), and microbially fermented feed and add ginseng polysaccharide just before use (MF + Gps group). Each group contained 5 pens per treatment and 20 birds per pen. The body weight and average daily gain in the Gps, MF, and MF + Gps groups increased significantly (*P* < 0.01), while the feed conversion ratio decreased significantly (*P* < 0.01). The combined use of MF and Gps showed a synergistic effect. There was no significant difference in villus height (cecal) between the experimental group and the Con group. The crypt depth of the three experimental groups exhibited a significantly lower value compared to the Control group (*P* < 0.05). The V/C ratio of the Gps group and MF + Gps was significantly increased (*P* < 0.05), but there was no significant difference in the MF group. Moreover, the diarrhea rate of the Gps and the MF + Gps groups was lower than that of the Con group, while that of the MF + Gps group decreased the mortality rate (*P* < 0.05). The serum tumor necrosis factor-alpha (TNF-α) and interleukin 6 (IL-6) levels in the MF, Gps, and MF + Gps groups decreased significantly (*P* < 0.01), the serum immunoglobulin G (IgG) levels increased significantly (*P* < 0.01), while the combination of MF and Gps had a synergistic effect. The combined use of Gps and MF not only further improved growth performance and immune parameters, but also reduced the diarrhea rate and mortality.

## Introduction

Microecological preparations, such as probiotics, prebiotics, synbiotics, as well as phytobiotics or phytochemicals, exhibit promising potential as diverse additives capable of enhancing the health and performance of poultry [1]. Although the advantageous impacts of numerous microecological preparations have been extensively documented, a prevailing perception exists that these products exhibit a lack of consistency [2, 3]. Also, combining microecological preparations with phytobiotics or phytochemicals may be more beneficial than using them separately [4]. An illustration of this can be seen in the potential of prebiotics to enhance the development and cloning of probiotic strains, while the combination of probiotics and phyto-genic feed additives may yield advantageous synergistic effects on the gut microbiota in juvenile chickens [4, 5]. Phytogenic feed additives (PFAs) are a wide range of bioactive compounds, which have potential positive effects on poultry health and productivity [4, 6]. The antioxidant components of PFAs belong to different chemical categories and can be recycled and obtained as plant extracts, essential oils, or resins [7]. *Panax* ginseng (*Panax* ginseng C.A. Meyer) contains a variety of active ingredients, such as polysaccharides, saponins, volatile oils, alkaloids, amino acids, and other chemical components, and most of the medicinal properties of ginseng are attributed to polysaccharides and ginsenosides [8–10]. The reported function of ginseng polysaccharide (Gps) is the enhancement of immune activity [9, 11]. Moreover, oral supplementation of Gps dosages (100, 200, and 400 mg/kg) eliminated weight loss in chickens following vaccination (H5N1) [12]. Gps also altered the composition and diversity of gut microbiota in chickens [13] and mice [14] with antibiotic-associated diarrhea, restored the gut microbiota, balanced metabolic processes, and promoted the recovery of the mucosa.

In poultry production systems, probiotics are used to improve feed efficiency and growth performance and reduce foodborne pathogens [15]. There are 12 strains approved in China that can be used to directly feed animals, including *Lactobacillus plantarum, Bacillus subtilis, Saccharomyces cerevisiae, Enterococcus faecium*, etc. Microbial fermented feed (MF) has gained significant popularity within the chicken industry due to its numerous benefits [16, 17]. The process of fermentation has been found to enhance the crude protein content, while simultaneously reducing the levels of crude fiber and anti-nutritional factors present in the feed [18]. These anti-nutritional factors include protease inhibitors, soybean protein, oligosaccharides in soybean meal, non-starch polysaccharides in corn, and phytic acid in bran. Consequently, the incorporation of fermented feed has been observed to positively impact the overall performance of chickens. Research reports that adding 7.5% fermented feed to the basal diet improves the growth performance, antioxidant capacity, and immune function of laying hens [19]. Feeding lactic acid bacteria fermented feed (supplement with lactic acid (160–250 mmol/kg feed) and acetic acid (20–30 mmol/kg feed)) can improve egg weight, shell weight, and shell hardness in laying hens [20]. Furthermore, providing a fermented diet has also been demonstrated to improve immune responses, antioxidant capacity, intestinal digestive function, and morphology, as well as the gut microbial ecosystem of broilers (*E. coli* and *Salmonella spp.* decreased linearly with probiotic fermented feed level) [21–23]. To date, the predominant focus of scholarly inquiry has been on the utilization of fermented feed or microorganisms as supplementary agents, leaving uncertain the viability of substituting conventional feed entirely with fermented feed. The Xuefeng black-bone chicken originated in Hongjiang City, Huaihua City, and Hunan Province, China. It is a kind of meat and egg chicken, a breed known for its high-quality meat. It has been officially recognized and included in the National Livestock and Poultry Genetic Resources Directory of China [13, 24, 25]. However, the Xuefeng black-bone chickens face challenges such as low slaughter weight, extended feeding cycle, and low feed conversion ratio (FCR), all of which significantly impact their economic profitability.

This study primarily investigates the potential synergistic effects of combining MF and Gps in promoting growth and reducing inflammation. Additionally, it explores the viability of exclusively utilizing fermented feed for complete feeding.

## Materials and methods

### Preparation of ginseng polysaccharide and microbially fermented feed

The Gps was extracted according to a literature procedure [26]. Briefly, the roots of *Panax* ginseng sourced from the Changbai Mountain in Huanren, Liaoning Province, China, were subjected to a triple decoction process using distilled water. All aqueous solutions were combined, concentrated under reduced pressure, and precipitated by adding 95% ethanol at 4 °C for 24 h to obtain crude Gps. Gps was further deproteinated by using the Sevag method and de-starch with a-amylase. Gps was prepared into a 5% solution with 1.5 mol/L NaCl, stirred at 50 °C for 4 h, and left at 4 °C overnight [9]. According to the phenol sulfuric acid method, the sugar content of ginseng polysaccharides is approximately 89%. Then, mix ginseng polysaccharides and feed with the quartering method.

Method of microbially fermented feed: dissolve the weighed brown sugar with water, then add the corresponding bacterial liquid according to the weight of the feed to be fermented (*S. cerevisiae* (5 × 10^7^ CFU/g, 250 mg/kg feed), *B. subtilis* (5 × 10^8^ CFU/g, 300 mg/kg feed), *L. plantarum* (3 × 10^8^ CFU/g, 1 ml/kg feed), and *E. faecium* (5 × 10^8^ CFU/mL, 1 ml/kg feed)), and left for 2 h to activate the bacteria. The ratio of full-price feed, brown sugar, and water is 100:1:35. Each time 200 kg of feed is fermented, the feed is poured in the sterilized basin, water and mixed bacterial liquid are added to the feed, and the shovel mixes evenly. Put the mixed feed into a 50 kg fermentation bag, seal it with a sealing machine, and then completely exhaust the air in the fermentation bag from the exhaust port, and then use a rope to tie the bag tightly. Finally, the bagged feed was pushed into the constant temperature incubator, the fermentation temperature was 35 °C, and the fermentation time was 10 days. The fermented feed is golden yellow, and it has a strong aroma of wine. *S. cerevisiae, B. subtilis, L. plantarum*, and *E. faecium* were obtained from the Guangdong Institute of Microbiology (Guangzhou, China).

### Experimental animals

A total of 400 Xuefeng black-bone hens at the age of 1 day were randomly assigned to four groups, each group contained 5 pens per treatment and 20 birds per pen: The feeding experiment was divided into four groups. Normal feed group (Control group), ginseng polysaccharide (Gps, 200 mg/kg) group, microbially fermented feed (completely replace the normal feed, MF) group, and microbially fermented feed and add ginseng polysaccharide just before use (MF + Gps group). The trial period consisted of 105 days, and the feed (pelleted) formulations for the two periods (0–4 weeks and 5–15 weeks) are shown in Table 1. The basal diet was formulated to meet the nutrient requirements suggested by the nutrient requirements of yellow chickens (NY/T 3645–2020). Water and feed were provided ad libitum. The hens were accommodated within a controlled environment, where the temperature and humidity levels were regulated between 25 and 35 °C and 60–70% respectively, through the utilization of the thermostat and fan. All chicks (0–4 weeks) were raised in individual cages with a wire mesh floor, and adult chickens (5–15 weeks) were reared individually in single cages. All hens had free access to drinking water and were fed at a fixed time every day in an enclosed chicken house under a standard lighting program of 16:8 h (light: dark). Excrement was collected once a day through conveyor belts. All the management procedures followed commercial settings, including normal immunization and disinfection, normal commercial feeding management, standard ventilation of the chicken house, and daily checking of birds. Feed was removed from the pen 24 h before sampling. The experimental animals (Xuefeng black-bone chickens) and the place were provided by Hunan Songyun Poultry Industry Co., Ltd., which is located at No. 12, Nanyue Mountain, Hongjiang District, Huaihua City, Hunan Province.

**Table 1:** Basic feed formulation and nutrient composition (air-dried basis, %)

Ingredient	Content (%)	
	Diet 1 (0–4 week)	Diet 2 (5–15 week)
Corn	59.0	59.0
Soybean meal	20.0	17.2
Cottonseed meal	3.8	8.5
Peanut meal	2.2	
Rapeseed meal	4.0	2.3
Fish meal	4.1	2.0
Wheat bran	0.6	5.0
Rapeseed oil	1.0	0
Calcium hydroxide	0.8	0.6
Stone powder	0.5	0.9
Premix	4.0	4.0
Salt	0.5	0.4
Nutritional composition		
Metabolizable energy (Mcal/kg)	12.46	12.70
Crude protein %	20.55	17.05
Calcium %	1.0	0.85
Available phosphorus %	0.45	0.4
Methionine%	0.51	0.42
Lysine %	1.1	1.11
Potassium %	0.85	0.71
Sodium %	0.2	0.19
Chlorine %	0.2	0.25

*Note*: The compound premix is provided for each kilogram of a basic diet with Cu 200 mg, Fe 1000 mg, Zn 1000 mg, Mn 1000 mg, Se 2.5 mg, I 15 mg, Ca 10%, total phosphorus 2.5%, sodium chloride 4–10%, leech 10%, choline 7500 mg, vitamin A 100,000 IU, vitamin D3 30,000 IU, vitamin E 850 IU, vitamin B1 30 mg, vitamin B2 130 mg, Vitamin B6 60 mg, vitamin B12 0.3 mg, strontium folate 22 mg, biotin 3 mg, niacin citrate 600 mg, barium citrate 45 mg, phytase 4000 U

### Determination of nutritional traits

The crude protein was estimated using the Kjeldahl method (GB/T6432-94). Crude fat was measured using the Soxhlet extraction method (GB/T6433-94). Ash content was determined by manual combustion at 850 °C (GB/T6438-94). The determination of moisture content refers to GB/T6435-94.

### Determination of diarrhea and mortality rate

When a chicken’s feces don’t take shape, we think it has diarrhea. The diarrhea was recorded daily during the initial feed formula (0–4 weeks). The diarrhea rate (%) = number of diarrhea chickens/total number of chickens * 100%. The mortality rate (%) = number of dead chickens/total number of chickens * 100%.

### Performance data and sample collection

To assess the growth performance of chickens, we monitored body weight (BW) at 0, 4, and 15 weeks, and feed intake from 5 to 15 weeks. Average daily feed intake (ADFI), average daily gain (ADG), and feed conversion rate (FCR) were calculated for 5–15 weeks by recording body weight and cumulative feed intake. On the 105th day of the trial, a total of 10 chickens per treatment were randomly chosen, with two chickens selected from each pen. The Blood collected from a chicken wing vein and euthanized with an overdose of CO_2_ [27]. The liver and spleen were immediately removed and weighed. Tissue samples of the cecal were obtained and gently flushed with 0.9% saline and fixed in 10% formalin for histomorphological analysis.

### Determination of blood biochemical parameters

As previously reported [28], the serum total protein (TP) (A045-2-2), albumin (ALB) (A028-2-1), total cholesterol (TC) (A111-1-1), and triglyceride (TG) (A110-1-1) were measured by an automatic biochemical analysis instrument (Olympus 2700, Japan). The reagent kit from Nanjing Jiancheng Biotechnology Institute.

### Detection of blood immune parameters

The serum tumor necrosis factor-alpha (TNF-α) (H052-1), interleukin 6 (IL-6) (H007-1), and immunoglobulin G (IgG) (H106-1) were measured by ELISA kit according to manufacturers from Nanjing Jiancheng Biotechnology Institute.

### Tissue samples of the cecal histomorphology

Five intestinal samples were randomly selected from each group and processed according to the method described by Thompson et al. [29]. In brief, the intestinal samples were collected from the distal (about 1 cm from the colon). Intestinal samples were dehydrated with increasing concentrations of ethanol, cleared with xylene (Surgipath Medical Industries, Richmond, IL), and embedded in paraffin wax (Thermo Fisher Scientific, Kalamazoo, MC). Cross-Sect. (5 μm) were stained with hematoxylin and eosin (GeneCopoeia, Rockville, MD). The stained sections were dehydrated with ethanol, cleared with xylene, and mounted with DPX mountant based on the method of Jiang et al. [30]. ImageJ software (National Institutes of Health, USA) was used to determine the morphometric measurements of villus height and crypt depth of the cecal using an Olympus BX40 F-3 microscope (Olympus Cooperation, Tokyo, Japan) attached to a digital video camera (Q-imaging, 01-MBF-200R-CLR-12, SN: Q32316, Canada) [31].

### Statistical analysis

The experimental design included a completely randomized design. Preliminary data processing using SPSS 17. The data are presented as mean ± standard deviation (SD) (table) or mean ± standard error (SEM) (figure). A Student’s t-test was used for a single variable comparison between two groups. One-way ANOVA followed by Tukey’s post hoc test and linear and quadratic regression [32] was used to examine interactions between multiple variables and a value of *P* ≤ 0.05 was considered to be a significant difference.

## Results

### Changes in nutrients in MF

Initially, we observed variations in the nutritional composition between the MF group and the Con group. As shown in Table 2, after microbial fermentation, crude protein (*P* < 0.05), crude ash (*P* < 0.05), and moisture (*P* < 0.05) in the feed were significantly increased compared with unfermented feed. However, crude fat was not significantly different.

**Table 2:** Changes in nutrients in MF (5–15-week, air-dried basis, %)^1^

	Control	MF
Crude protein	17.04^b^ ± 0.21	18.38^a^ ± 0.18
Crude fat	3.56 ± 0.10	3.71 ± 0.12
Crude ash	6.66^b^ ± 0.15	7.77^a^ ± 0.13
Moisture	13.07^b^ ± 0.20	35.07^a^ ± 0.68

A Student’s t-test was performed. In the same row, values with different letter superscripts mean a significant difference (*P* < 0.05). Data are presented as mean ± SD

^1^Feed intake is calculated according to the dry matter content

### Growth performance

As presented in Table 3, the BW of the Gps, MF, and MF + Gps groups were not significantly different from the Con group in the fourth weeks. However, the BW of the Gps, MF, and MF + Gps groups at week 15 was significantly higher than that of the control group (*P* < 0.01). And there was a significant increase in ADG in the Gps, MF, and MF + Gps groups compared to the Control (*P* < 0.01). Additionally, there was a significant decrease in FCR (*P* < 0.01). There was no significant difference in the ADFI among all groups. The combined utilization of MF and Gps demonstrated a notable synergistic effect, surpassing the individual effects of MF or Gps in terms of promotion.

**Table 3:** Effects of MF and Gps on growth performance of Xuefeng black-bone chickens^1^

Item^2^	Treatment^3^				*p*-value		
	Control	Gps	MF	MF + Gps	*P*_ANOVA_	*P*_Linear_	*P*_Quadratic_
Initial BW (g)	33.49 ± 2.50	33.64 ± 3.19	33.23 ± 3.58	33.74 ± 3.13	0.793	0.718	0.609
BW (g, 4 weeks, diet 1)	171.75 ± 33.30	172.20 ± 29.97	170.15 ± 36.08	177.56 ± 25.45	0.306	0.163	0.260
BW (g, 15 weeks, diet 2)	796.67^b^ ± 36.68	910.42^a^ ± 42.44	943.90^b^ ± 67.95	951.85^a^ ± 28.27	< 0.001	< 0.001	0.005
ADFI (g, 1–15 weeks)	24.48 ± 1.24	24.66 ± 0.27	23.20 ± 1.14	23.51 ± 0.34	0.998	0.891	0.970
ADG (g, 1–15 weeks)	8.13^b^ ± 0.37	9.29^a^ ± 0.43	9.63^a^ ± 0.69	9.71^a^ ± 0.29	< 0.001	< 0.001	0.005
FCR (1–15 weeks)	3.03^a^ ± 0.15	2.65^b^ ± 0.03	2.59^b^ ± 0.13	2.54^b^ ± 0.04	< 0.001	< 0.001	0.005

One-way ANOVA was performed. In the same column, values with different letter superscripts mean a significant difference (*P* < 0.05). Data are presented as mean ± SD

^1^Feed intake is calculated according to the dry matter content

^2^BW = bodyweight; ADFI = average daily feed intake; ADG = average daily gain; FCR = feed conversion ratio

^3^Gps = normal feed and ginseng polysaccharide (200 mg/kg) group (Gps group); MF = microbially fermented feed group, MF + Gps = microbially fermented feed and add ginseng polysaccharide (200 mg/kg) just before use

### Cecal morphology

**Fig. 1:**
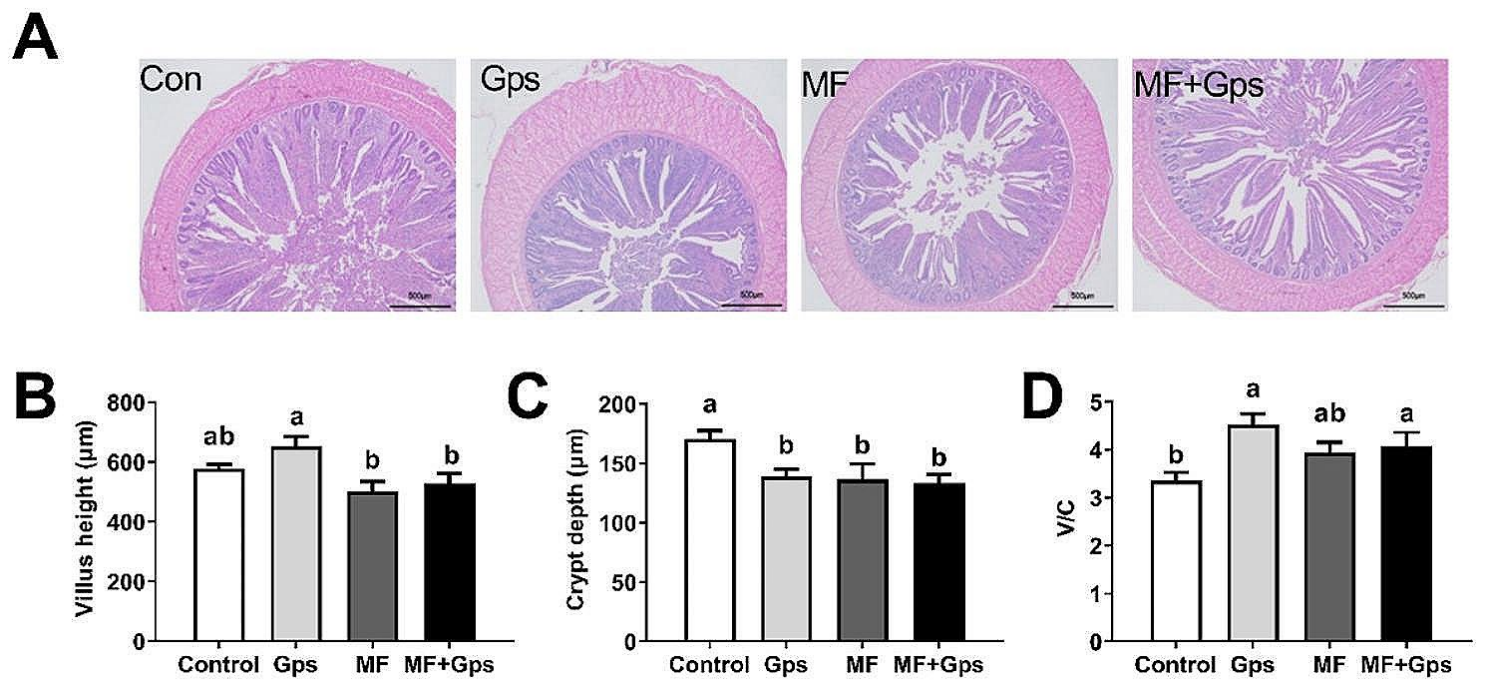
Effects of MF and Gps on cecal morphology in Xuefeng black-bone chickens. (**A**) Morphologic analysis of cecum. Scale bars: 500 μm. (**B**–**D**) The cecum villus length (**A**), crypt depth (**B**), and V/C ratio (**C**) in Xuefeng black-bone chickens. One-way ANOVA was performed, and values with different letter superscripts mean significant difference (*P* < 0.05). Data are presented as mean ± SEM

The cecal morphology of the Control, Gps, MF, and MF + Gps groups is depicted in Fig. 1A. There was no significant difference in villus height between the experimental group and the Con group (Fig. 1B). The crypt depth of the three experimental groups exhibited a significantly lower value compared to the Control group (*P* < 0.05), with no significant difference observed among the experimental groups themselves (Fig. 1C). Therefore, compared with the Control group, the V/C of the Gps group and MF + Gps was significantly increased (*P* < 0.05), but there was no significant difference in the MF group (Fig. 1D). Results show the promoting effect of Gps group on V/C was better than that of MF + Gps group.

### Diarrhea rate and mortality

As shown in Table 4, the diarrhea rate of the Gps and the MF + Gps groups was significantly (*P* < 0.05) lower than that of the Control group. Conversely, the MF group and the Con group exhibited similar rates of diarrhea. In the overall period, the MF + Gps group showed a significantly (*P* < 0.05) decreased mortality rate than that of the Con group.

**Table 4:** Effects of MF and Gps on diarrhea rate and mortality of Xuefeng black-bone chickens

Item	Treatment				*p*-value		
	Control	Gps	MF	MF + Gps	*P*_ANOVA_	*P*_Linear_	*P*_Quadratic_
Diarrhea rate	7.69%^a^±0.52%	5.77%^b^±0.34%	7.69%^a^±0.47%	3.85%^c^±0.36%	< 0.001	< 0.001	< 0.001
Mortality rate	5.85%^a^±0.44%	5.69%^a^±0.49%	5.73%^a^±0.59%	4.77%^b^±0.54%	0.016	0.007	0.104

One-way ANOVA was performed. In the same column, values with different letter superscripts mean a significant difference (*P* < 0.05). Data are presented as mean ± SD

### Serum biochemical parameters

As presented in Table 5, there was no significant difference in the TP, ALB, and TG among the four groups. Additionally, compared with the Con group, the TC in the Gps group decreased significantly (*P* < 0.05), while there was no significant difference between the MF and the MF + Gps groups.

**Table 5:** Blood biochemical parameters of Xuefeng black-bone chickens

Item^1^	Treatment				*p*-value		
	Control	Gps	MF	MF + Gps	*P*_ANOVA_	*P*_Linear_	*P*_Quadratic_
TP (g/L)	49.10 ± 5.08	46.75 ± 5.76	46.74 ± 3.81	49.63 ± 1.19	0.726	0.883	0.267
ALB (g/L)	14.44 ± 2.31	13.32 ± 2.24	14.30 ± 1.52	16.75 ± 1.60	0.161	0.220	0.094
TC (mmol/L)	3.66^a^ ± 0.55	2.67^b^ ± 0.41	3.71^a^ ± 0.57	3.28^ab^ ± 0.36	0.019	0.072	0.273
TG (mmol/L)	11.99 ± 0.88	11.42 ± 0.66	11.47 ± 0.66	11.34 ± 0.87	0.670	0.305	0.594

One-way ANOVA was performed. In the same column, values with different letter superscripts mean a significant difference (*P* < 0.05). Data are presented as mean ± SD

^1^TP = total protein; ALB = albumin; TC = total cholesterol; TG = total triglyceride

### Immune parameters and organs

**Fig. 2:**
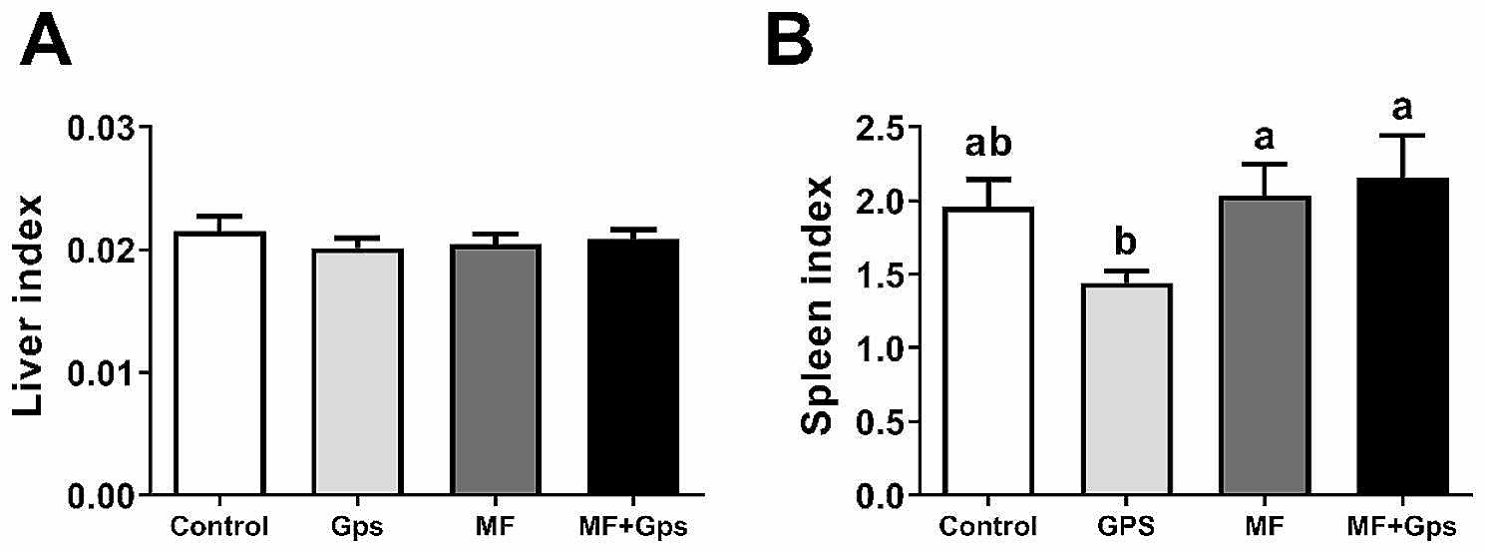
Effects of MF and Gps on immune organs of Xuefeng black-bone chickens. (**A**, **B**) The liver and spleen index of Xuefeng black-bone chickens (n = 15, each group contained 5 pens per treatment and 3 chickens per pen). One-way ANOVA was performed, and values with different letter superscripts mean significant difference (*P* < 0.05). Data are presented as mean ± SEM

As shown in Table 6, compared with the Control group, the serum TNF-α and IL-6 levels in the MF, Gps, and MF + Gps groups decreased significantly (*P* < 0.01), while the serum IgG levels increased significantly (*P* < 0.01). Importantly, the combination of MF and Gps had a synergistic effect in reducing serum TNF-α concentration and increasing IgG level, with the level of IL-6 being the same as that in the Gps group. Moreover, there was no significant difference in the liver index among the four groups (Fig. 2A). Compared with the Con group, the spleen index in the Gps group decreased significantly (*P* < 0.05), while there was no significant difference in the MF group and the MF + Gps group (Fig. 2B).

**Table 6:** Immune parameters in the serum of Xuefeng black-bone chickens in each treatment group

Item	Treatment				*p*-value		
	Control	Gps	MF	MF + Gps	*P*_ANOVA_	*P*_Linear_	*P*_Quadratic_
IL-6 (ng/L)	15.72^a^ ± 0.84	14.15^c^ ± 0.79	14.91^b^ ± 0.74	14.25^c^ ± 0.20	< 0.001	< 0.001	< 0.001
TNF-α(pg/L)	243.81^a^ ± 27.94	184.87^b^ ± 22.94	179.03^b^ ± 27.40	170.16^b^ ± 22.35	< 0.001	< 0.001	0.021
IgG(µg/L)	1.34^c^ ± 0.25	2.03^b^ ± 0.22	2.15^b^ ± 0.17	2.61^a^ ± 0.12	< 0.001	< 0.001	< 0.001

One-way ANOVA was performed. In the same column, values with different letter superscripts mean a significant difference (*P* < 0.05). Data are presented as mean ± SD

## Discussion

The reported functionality of Gps includes immune enhancement and antioxidation [9, 11]. As a feed additive, it can eliminate the weight loss of chickens inoculated with H5N1 [12] and improve jejunum morphology and microbiota composition [13]. MF has been widely used in the chicken industry [16, 17]. Prior research has demonstrated that the implementation of MF is regarded as a beneficial approach in the livestock industry and is extensively employed. It has the potential to enhance various aspects such as production performance, antioxidant capacity, immune function, intestinal digestive capacity, intestinal morphology, and intestinal microbial ecosystem of both laying hens [18–20] and broilers [21–23]. This study primarily investigates the potential synergistic effects of combining MF and Gps in promoting growth and reducing inflammation.

The utilization of MF can enhance the breakdown of complex macromolecular nutrients present in animal feed, thereby augmenting animals’ digestive and absorptive capacities, and ultimately fostering growth [33–35]. Before the commencement of the feeding experiment, we conducted a nutritional assessment comprising of control group and the MF group. The results showed that after microbial fermentation, the nutritional value of feed was improved. It was found that the Gps group, MF group, and MF + Gps group significantly improved growth performance. More importantly, the combined use of MF and Gps showed a good synergistic ability in promoting growth. In addition, we also found that MF, Gps, and MF + Gps did not significantly improve the growth performance of chickens during the brooding period. Fermentation feeds have been shown to improve digestion and absorption, thereby improving chicken production [13, 36–38]. The small intestine serves as the primary site for poultry to undergo digestion and absorption of nutrients, with its functionality being intricately linked to the dimensions of villus height, crypt depth, and the ratio between villus height and crypt depth [39]. The cecum serves as a significant site for fermentation in avian species, while also possessing a distinct capacity for nutrient absorption [39]. Our previous study reported that feeding MF or Gps significantly increased the jejunal villus height, decreased the crypt depth, and increased the V/C ratio, with the combined use of MF and Gps showing a stronger effect [13]. In terms of cecal morphology, MF, Gps, and MF + Gps can significantly reduce crypt depth and increase the V/C ratio. Although the combined use of MF and Gps did not achieve a better effect, it was at the same level as Gps alone. We speculate that Gps altered the composition and diversity of gut microbiota restored the gut microbiota, and promoted the intestinal development.

During the feeding experiment, it was observed that the inclusion of Gps resulted in a significant reduction in the occurrence of diarrhea in Xuefeng black-bone chickens, with a decrease of 24.97%. Conversely, the addition of MF did not exhibit the same effect. Furthermore, the concurrent utilization of MF and Gps yielded a notable reduction in the prevalence of diarrhea (by 49.93%) and mortality rates (by 18.46%). As our previous study found, the combined use of MF and Gps significantly increased the abundance of *Sutterella* in the jejunum and decreased the abundance of *Asteroleplasma* [13]. And, a previous study reported that the members of the genus *Sutterella* were inversely associated with diarrhea in mice [40]. In addition, *Sutterella* is widely prevalent in the human gastrointestinal tract, and its deficiency alters the colonic microbiota, leading to the disruption of normal colonic epithelial cell function and inflammatory bowel disease [41]. Therefore, whether *Sutterella* is involved in the regulation of diarrhea in chickens requires further research.

A combined effect of MF and Gps on the immune function of Xuefeng black-bone chickens was also examined in this study. The combined use of Gps and MF does not exhibit any significant impact on serum biochemical indicators, except for the potential reduction of TC levels when ginseng polysaccharides are added. TNF-α and IL-6 are important pro-inflammatory cytokines, the elevated concentration in serum is usually related to some diseases and is regarded as a marker for inflammation [42, 43]. IgG is one of the most abundant antibodies in the serum, which has the immune regulation function of antibacterial and antiviral [44]. The findings of our study indicate that the serum levels of TNF-α and IL-6 were significantly reduced in the MF group, Gps group, and MF + Gps group when compared to the Con group. Additionally, there was a significant increase in serum IgG levels. More importantly, the combination of MF and Gps had a synergistic effect in reducing serum TNF-α concentration and increasing IgG level, thereby indicating a stronger elevation of immune capacity. The spleen plays a crucial role in both nonspecific and specific immunity, and the measurement of the spleen index serves as an indicator of immune function and prognosis. Our findings indicate that the addition of Gps to feed resulted in a significant decrease in spleen index. However, the addition of MF or the combination of feeding MF with Gps did not exhibit any notable impact on the spleen index. There are studies available that provide evidence of the decrease in thymus and spleen indices following the administration of polysaccharide treatment in mice [45]. Furthermore, previous studies have reported that both Gps and MF have immune-enhancing effects [9, 11, 19]. Based on the findings of our study, it is justifiable to hypothesize that the administration of ginseng polysaccharides may enhance spleen functionality, despite the observed decrease in spleen index.

## Conclusions

In conclusion, the utilization of Gps or MF can augment the growth performance and immune indices of Xuefeng black-bone chickens. Gps can also improve intestinal development. Moreover, the combined utilization of Gps and MF demonstrates a synergistic effect, leading to further improvements in growth performance and immune indices. Additionally, it effectively mitigates the incidence of diarrhea and mortality, outcomes that cannot be achieved when Gps or MF are used individually. Consequently, our study has successfully devised a compound feed formulation that effectively enhances the growth performance, intestinal development, and immune parameters of chickens.

## Data Availability

The datasets used and/or analyzed during the current study are available from the corresponding author upon reasonable request.

## Abbreviations

PFAs: Phytogenic feed additives
Gps: Ginseng polysaccharide
FCR: feed conversion ratio
MF: Microbial fermented feed
ADFI: Average daily feed intake
ADG: average daily gain
TP: total protein
ALB: albumin
TC: total cholesterol
TG: total triglyceride
BW: body weight
TNF-α: tumor necrosis factor-alpha
IL-6: interleukin 6
IgG: immunoglobulin G
